# Assessment Tools for Health Literacy among the General Population: A Systematic Review

**DOI:** 10.3390/ijerph15081711

**Published:** 2018-08-10

**Authors:** Hongyan Liu, Huan Zeng, Yang Shen, Fan Zhang, Manoj Sharma, Weiyun Lai, Yu Zhao, Genhui Tao, Jun Yuan, Yong Zhao

**Affiliations:** 1 School of Public Health and Management, Chongqing Medical University, Chongqing 400016, China; 15178844252@163.com (H.L.); zenghuan586@aliyun.com (H.Z.); cqmusssy1989@163.com (Y.S.); 102715@cqmu.edu.cn (F.Z.); 2 Research Center for Medicine and Social Development, Chongqing Medical University, Chongqing 400016, China; 3 Collaborative Innovation Center of Social Risks Governance in Health, Chongqing Medical University, Chongqing 400016, China; 4 Behavioral & Environmental Health, School of Public Health, Jackson State University, Jackson, MS 39213, USA; manoj.sharma@jsums.edu; 5 Health Sciences, Walden University, Minneapolis, MN 55401, USA; 6 Project Office, Chongqing Health Education Institute, Chongqing 400016, China; m13372713201@163.com (W.L.); zy10550812@163.com (Y.Z.); 18166310077@163.com (G.T.)

**Keywords:** health literacy, health equality, health in all policy, assessment tool, general population

## Abstract

Health literacy is an important determinant of health, and is one of the key indicators of a healthy city. Developing and improving methods to measure health literacy is prudent and necessary. This review summarizes the findings of published tools for assessing health literacy among the general population to provide a reference for establishing health literacy assessment tools in the future. In this systematic review, PubMed, Embase, and Web of Science were used to search articles regarding tools for assessing health literacy among the general population published up to 10 January 2018. Two researchers independently conducted literature screening, quality assessment of methodology, and data extraction according to preset inclusion and exclusion criteria. The quality assessment of the research was examined with the use of the specifications of the reporting guidelines for survey research (SURGE). Eleven articles met the inclusion criteria. All included instruments in monitoring the health literacy of the general population were presented through the form of questionnaires. The multistage process of making all the scales generally involved the following steps: item development, pre-testing, and evaluation of readability. However, the specific methods were different. Internal consistency for all the instruments was acceptable but with weak consistency among the subscales for some instruments. Most of the identified instruments derived from the definition of health literacy or were based on existing health literacy theory. Approximately 30% of the performed studies provided no description of the important features specified in the SURGE. This review indicates a trend in the increasing tools for assessing the health literacy of the general population by using multidimensional structures and comprehensive measurement approaches. However, no clear “consensus” was observed in the dimensions of health literacy tools.

## 1. Introduction

Population health is subject to social, economic, environmental, personal characteristics, personal behavior, and other factors. Moreover, health literacy is among the critical determinants of health [1]. Health literacy was conceptualized as “the cognitive and social skills which determine the motivation and ability of individuals to gain access to, understand, and use information in ways that promote and maintain good health” [2]. More and more countries have monitored and assessed the health literacy among adults. The 2003 National Assessment of Adult Literacy found that 14% of America’s adults had low health literacy [3]. In 2006, the Australian Bureau of Statistics revealed that approximately 60% of Australian adults had low health literacy [4]. Health literacy monitoring results among Chinese residents in 2012 showed that about 91.2% of Chinese residents had low or insufficient health literacy [5]. The first European survey on health literacy found that 47% of the populations of eight European countries had low health literacy [6]. Low or insufficient health literacy has become a worldwide public health problem.

Improving people’s health literacy is one of the most fundamental, economic, and effective measures to improve the health level of the entire population [7]. First, improving health literacy is a significant cause of enhancing a complex set of health-related results. Prior studies showed that health literacy plays an important part in women’s reproductive health [8], chronic kidney disease [9], asthma and chronic obstructive pulmonary disease [10], human immunodeficiency virus [11], and sleeping hours in children [12], and may impact behaviors and outcomes. Second, improving health literacy may reduce health inequities. According to the 9th Global Conference on Health Promotion in 2016, improving the health literacy of the general public provided the foundation for citizens who could successfully participate in community action for health and governments who assumed liabilities of resolving health equity [13]. Third, improving health literacy is an advantage for mutually reinforcing other literacies, because health literacy often intersects with educational, legal, financial, technological, and other forms of literacy [14].

Given the importance of health literacy, developing comparable and reliable measurement tools for assessing health literacy among the population is prudent. A World Health Organization (WHO) report on the roles for stakeholders in advancing health literacy mentioned that assessment tools for health literacy should be developed and improved by the research institutions [14]. To date, various frameworks or scales are available for assessing and measuring health literacy based on different subjects, diseases, or theoretical foundations, such as the development of the health literacy instrument for female marriage immigrants [15], development of the digital health literacy instrument [16], conceptualizing a new approach to adolescent health literacy [17], health literacy tools in the outpatient setting [18], and the development of a hypertension health literacy instrument [19]. However, no reliable, definitive, and comparable health literacy scale exists for a global population.

### Aims

In this systematic review, published tools for assessing health literacy among the general population were selected, analyzed, and summarized. The following categories usually need to be considered in a review: health literacy measurement approaches and modes; theoretical basis; specific dimensions or items; analysis of the feasibility, reliability, and validity of the scale; and the findings of the identified tools. Therefore, in view of the limited evidence on how to develop and validate assessment tools for the health literacy among the general population, the present review aimed to assess the status of tools for the assessment of health literacy from several perspectives and to provide some suggestions for establishing health literacy assessment tools in the future.

## 2. Materials and Methods

### 2.1. Retrieval Strategy

Three electronic databases (PubMed, Embase, and Web of Science) were accessed for published articles and other publications reporting on tools for the assessment of the health literacy of the general population. The keywords “health literacy” were used. The accessed studies were published up to 10 January 2018. The reference lists for published articles were searched to ensure the comprehensiveness of the included articles.

### 2.2. Inclusion and Exclusion Criteria

Published studies were considered appropriate for the literature review when they met the following criteria: (1) developing and validating the assessment tools for the health literacy; (2) the general population (aged >15 years) for the investigation or application objects of the tool; and (3) the preceding two points were not subject to geographic regions or languages.

Studies were excluded when the four factors were determined: (1) repeat verification of the same tool; (2) patients or a special group of other people were the application objects of the tool; (3) review studies; and (4) no available full text.

### 2.3. Literature Screening, Quality Assessment, and Data Extraction

**Figure 1:**
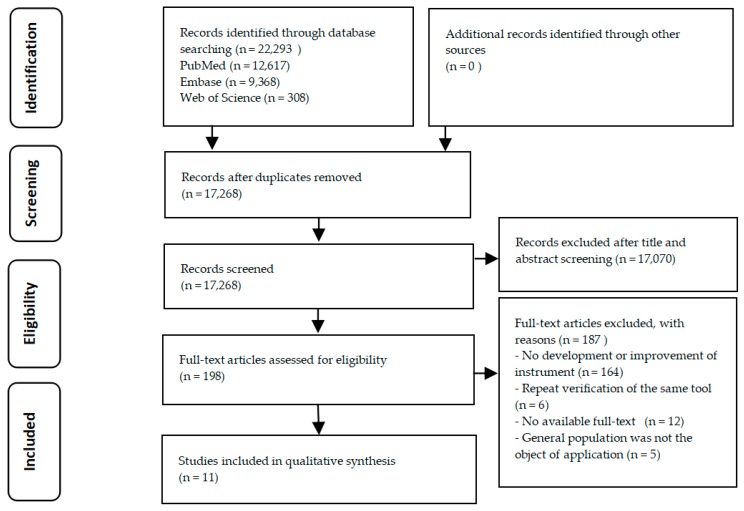
Preferred Reporting Items for Systematic Reviews and Meta-Analysis (PRISMA) flow diagram of literature selection. *n:* number of articles.

The studies were screened according to the Preferred Reporting Items for Systematic Reviews and Meta-Analysis (PRISMA) flow diagram (Figure 1) [20]. According to the preset inclusion and exclusion criteria, literature screening, quality assessment of methodology, and data extraction were conducted by two researchers separately. Additionally, the filtered information was cross-checked by the two authors to ensure the effectiveness and integrity of the data. If the opinions of the two researchers were not uniform, it was resolved by discussing or referring to the opinions of third parties (including three authors: Jun Yuan, Weiyun Lai, and Yong Zhao). The quality assessment of the research was examined with the use of the specifications of the reporting guidelines for survey research (SURGE), which was captured in eight domains [21]. When some specific items within the eight domains were unrelated to the assessment of the health literacy index, these items were adjusted and modified. Therefore, the appropriate reporting guidelines for health literacy indices ultimately included 30 items. The data were extracted in the following categories: first author, publication year, national sources, theoretical basis, methods, sample, domains, items, feasibility, and findings.

## 3. Results

In this review, the PRISMA flow diagram in Figure 1 shows the process of exclusion and inclusion. Eleven studies conformed to the inclusion criteria, including 11 tools for the assessment of health literacy among the general population. The majority of excluded studies (*n* = 164) did not add to the development or improvement of health literacy tools among the general population. Some of the studies (*n* = 12) were excluded because the full text was not available (e.g., incomplete data extraction). Five instruments [22,23,24,25,26] had to be excluded because their object of application was not the general population (e.g., patients). Six excluded instruments [27,28,29,30,31] were direct translations of already-developed instruments in several languages or were repeat verification of the same tool.

### 3.1. Main Instrument Characteristics

Table 1 shows the main instrument characteristics and data related to all the identified studies. The 11 studies from nine countries (the USA, China (Taiwan), the U.K, Australia, Netherlands, Japan, Iran, Thailand, and Zambia) were included, and the main features of the tool were as follows: scale names, theoretical basis, methods, sample, domains, items, feasibility, and findings. All identified instruments for measuring and monitoring health literacy among the general population were presented in the form of questionnaires and were self-reported. Thus, these measurements were subjective. The multistage process of making all the scales generally involved the following steps and methods: (1) item development (expert consultation, an iterative process, in-depth interviews, real-world health-related stimuli, or comprehensive review of the literature); (2) pre-testing (participant feedback, questionnaire, internet-based information seeking, or interviews); and (3) evaluation of readability (factor analysis methods, scale score, and reliability calculation, or causal models for measuring health literacy). All instruments used a multidimensional concept of health literacy. According to the dimensions of the instrument, all instruments can be divided into three categories: (1) measurement modes based on health-related fields; (2) measurement modes based on health-related abilities, including accessing, understanding, appraising, and applying health-related information; and (3) the combination of both measurement modes.

### 3.2. S Measurement Modes Based on Health-Related Fields

Two instruments (the public health literacy knowledge scale (BHLKC) and the mental health literacy scale (MHLS)) used measurement modes based on health-related fields and were published as of 10 January 2018. Based on the “Thirteen Essential Facts for Life Messages”, the BHLKC from the United States included 13 topics (Table 1) and was comprised of 17 items (Supplementary Materials S1). The Spearman’s rho correlation between the BHLKC and the science literacy scale with *r* = 0.391 was acceptable, indicating different constructs of health literacy. The studies of the BHLKC signified that the differences in perspective between health literacy at an individual level and at the level of public health constituted important challenges in evaluating health literacy. On the basis of Jorm’s seven components of mental health literacy, the MHLS from Australia included seven domains (Table 1), and was comprised of 35 items (Supplementary Material S1). The main findings of the MHLS were that this scale might enable efficient identification of individuals who have low levels of mental health literacy. Both approaches measured and monitored topics in specific scale fields, demonstrating an acceptable internal consistency with a Cronbach’s alpha of 0.797. The BHLKC and the MHLS may both serve as one component of a complete measure of health literacy. Table 1 shows the detailed features of the instruments based on health-related fields.

### 3.3. Measurement Modes Based on Health-Related Abilities

By using measurement modes based on health-related abilities, six instruments (Zambia’s health literacy scale (ZHLC), the health literacy management scale (HeLMS), the Health Literacy Skills Instrument (HLSI), the Iranian Health Literacy Questionnaire (IHLQ), the All Aspects of Health Literacy Scale (AAHLS), and the 14-item health literacy scale (HLS-14)) basically applied the necessary capabilities of obtaining, understanding, processing health-related information, and decision-making in different manners or with different questions. The ZHLC is a 15-item tool that reflects four abilities with the use of simple and representative indicators. The ZHLC demonstrated acceptable internal consistency, with a Cronbach’s alpha of 0.68 and good content validity. The ZHLC was a simple and representative health literacy indicator based on the Institute of Medicine’s definition, and had a large national sample. The HeLMS, HLSI, and IHLQ extended or supplemented other abilities on the basis of four basic abilities: communication skills, assessment skills in virtual media, computations skills, and social support. Three tools had a relatively large number of items (29/25/36) and acceptable internal consistency. The innovation point of HeLMS is that it evaluates potentially variable factors of these abilities. The HLSI assesses a series of skills that individuals often need to monitor and manage their health during health and illness. The IHLQ originated from Iranian culture. Thus, it can efficiently and accurately measure different aspects of Iran’s health literacy. The AAHLS and HLS-14 measure functional, communicative, and critical health literacy by using 14 items and were adapted from Nutbeam’s health literacy theory and Ishikawa and colleagues’ health literacy scale specific to diabetes patients. The relevant items of the AAHLS and the HLS-14 were partially modified and supplemented using different methods, demonstrating good internal consistency and consistency among the subscales, except for weak consistency among the subscales for the AAHLS. The AAHLS indicated how to transform the Nutbeam framework into a practical measurement method and thus contributed to further improving the concept of health literacy. The HLS-14 may be especially beneficial in health education and promotion for locals. Table 1 shows the detailed features of the instruments based on health-related abilities, and Supplementary Material S1 (Table) illustrates the detailed items of the instruments.

### 3.4. Combination of Both Measurement Modes

Three instruments (the Mandarin Health Literacy Scale (MaHLS), the European Health Literacy Survey Questionnaire (HLS-EU-Q), and the ABCDE (alcohol, baccy, coping, diet, and exercise)-health literacy scale (ABCDE-HLS)) combined both measurement modes. The MaHLS combines four kinds of abilities and different health-related fields. These different health-related fields include the Institute of Medicine’s definition of health literacy, and six main field types (Table 1). The MaHLS had a high overall internal consistency (Cronbach’s alpha = 0.97) and good predictive validity. The method described in MaHLS could be used to develop and assess health literacy scales for other language speakers. The HLS-EU-Q has 47 items (took 20–30 min) based on four basic abilities and three health-related fields (Table 1). The HLS-EU-Q had an accepted consistency among the subscales of Cronbach’s alpha of 0.51–0.91. The HLS-EU-Q captured a wide public health perspective. The ABCDE-HLS with an accepted consistency among the subscales of Cronbach’s alpha of 0.611–0.912 has 64 items based on four basic abilities and four domains (alcohol, baccy, coping, diet, and exercise). The ABCDE-HLS is likely to be useful in surveys, intervention evaluation, and studies of the needs and abilities of individuals. Table 1 shows the detailed features of the instruments by using the combination of both measurement modes, and Supplementary Material S1 displays the detailed items of the instruments.

### 3.5. Quality Assessment of Health Literacy Instrument Studies

The quality assessment of the survey studies on measuring health literacy is presented in Table 2. Among the 11 included articles, approximately one-third provided no description of the important features specified in the SURGE. Eleven of the 30 reporting items were appropriately described in all identified articles (Table 2). Moreover, the description of the Methods section of all published studies was limited. The method of questionnaire administration, dates of data collection, description of methods for replication, and methods for data entry not described were in 90.9%, 54.5%, 18.2%, and 81.8%, respectively. A total of 36.4% did not describe the scoring methods of tool, and 36.4% did not describe the limitations among the performed studies. Furthermore, the description of specific items regarding sample selection, response rates, and ethics and disclosure all failed to reach 100% among the features of all publications.

## 4. Discussion

The significance of improving health literacy has been realized by several countries and individuals in recent years. To our knowledge, this systematic review was the first to explore the assessment tools for health literacy among the general population. In our review, the health literacy assessment tools were developed in the past 10 years. We mainly considered the status quo of the existing measuring instruments for health literacy on the basis of theoretical basis, design methods, specific dimensions or items, and reliability analysis. In addition, this review put forward some suggestions for enriching the knowledge and direction of health literacy scales.

First, the tools for measuring and monitoring health literacy came from many countries, and the same tool may have been translated into different language versions, such as the HLS-EU-Q’s translation from English to the language of the other six countries [41]. Therefore, the constraints of language may no longer exist with regard to the assessment of health literacy. This condition further indicated the possibility of establishing an internationally comparable and reliable health literacy instruments among populations in the future.

Second, we found that most studies used an underpinning theory of health literacy. Whether this theoretical basis can be completely suitable for this instrument and whether some items in this instrument can represent a dimension were worthy of investigation. These factors needed further consideration and a clear definition. After all, our purpose was not to develop the basic theory of health literacy in the studies [43]. Although an overall reliability of Cronbach’s alpha for all instruments were acceptable, the reliability of part of the scales [34,38,41,42] had yet to be modified and improved to achieve a satisfactory range of 0.80–0.90. These concerns were the dynamic issues in the development and validation of the scales. Therefore, dynamic health literacy models should be considered when developing new tools in the future to improve measuring tools.

Third, we found that two measuring tools [32,33] were aimed separately at one field of health literacy. The fields areas of health literacy had been explored as follows: Digital Health Literacy Instrument [16], Hypertension Health Literacy Assessment Tool [19], and Oral Health Literacy Instrument [44]. Given that these measurement modes may serve as one component of a complete measure of health literacy, measurement scales for health literacy may also be further developed and validated in other areas, such as nutrition and environment.

Moreover, we found that most of the instruments applied a multidimensional measurement of health literacy among the general population, mainly through abilities such as obtaining, understanding, and processing health-related information, as well as decision-making. These abilities were principally derived from the definition of health literacy. With the rapid growth of the field of health literacy, some changes in the definition of health literacy lacked common views [43]. Meanwhile, the concept of health literacy was constantly expanded and explored from different perspectives [45,46,47]. In this review, recent studies have added several other expanded abilities, such as assessment skills in virtual media and social support [37,42], which were lacking in the previous review of health literacy measurement tools [48]. These observations showed that the newly developed tools had consistently made up for previous deficiencies. In addition, except for an article on a tool to develop mental health literacy alone, mental health skills had not yet been integrated into all the assessment tools for the health literacy among the general population. This challenge was also emphasized in previous review studies [49]. These factors may indicate that the various evolving aspects of health literacy will require the development of new measuring tools to fully confirm health literacy among the general population.

Furthermore, we found that all tools for monitoring health literacy adopted a questionnaire approach. Self-reported monitoring and measurement tools for health literacy are subjective in questionnaire investigation. For data collection and participant feedback, we suggest that other methods should be combined with these subjective methods, such as interviews and observations, to increase the authenticity and objectivity of the data. Meanwhile, a comprehensive approach to health literacy may include both clinical and public health approaches to solve the problem of the differences in perspective between health literacy at an individual level and at the level of public health [32]. In this review, the objects of the interview came from a few fields and mainly included epidemiologists; specialists in oral health; specialists in community medicine; health service managers and commissioners; health workers and local service users; and health care, education, and psychometrics experts. Public health is an important area for implementing health literacy, but other factors—scientific, cultural, and social—were also closely related to health literacy [43]. The opinion of an expert or person is an important factor of specific items of the measurement tools to improve the comprehensiveness of health literacy assessment tools. Therefore, when developing and validating new tools, it is necessary to carefully consider the diversity and expertise of the interviewees.

Finally, the report guide was a useful way to assess the quality of the original research in a systematic review. However, no validated and comprehensive guidelines for reporting surveys currently exist [21]. In our review, we presented that the description frequency of the reported items was different in some dimensions and items of the SURGE. In particular, the four parts of methods, namely, response rates, sample selection, ethics, and disclosure, need to be emphasized and adequately described, consistent with the previous work. These factors indicated that the comprehensiveness of the study on health literacy assessment tools should be given considerable attention.

Therefore, the development and improvement of health literacy assessment tools for the general population should mainly include the following considerations: First, the representativeness and comprehensiveness of the theoretical basis and dimensions of health literacy are the primary considerations for developing assessment tools. However, the theory and dimensions of health literacy are still in the exploratory stage. Second, experts or individual interviewees are also important factors in developing assessment tools, but the current interviewees’ professional field is not sufficiently comprehensive. In addition, the monitoring method of health literacy is also part of determining whether the assessment tool is applicable. The monitoring methods of health literacy should have certain objectivity, but the current health literacy monitoring methods are more of a questionnaire survey, which has certain subjectivity. Finally, the dynamics of assessment tools for the health literacy should also be considered. For example, the establishment of a health literacy monitoring database is conducive to real-time updating of data so as to apply to the development of health literacy.

This review has certain limitations. First, the retrieval source included only three databases when retrieving literature in our review, and we may have missed some relevant articles. Moreover, the quality assessment of the articles was examined using the SURGE. However, because there was no scoring scheme regarding the SURGE, we qualitatively analyzed the results of the quality assessment of the included articles. Finally, a few parts of the content and characteristics in the health literacy instruments (e.g., the specific item, reliability, and validity) were not always available despite making attempts through the search from other channels and resources for help. Apart from this, this review has some notable strengths as well. First, we followed the corresponding guidelines when screening the literature, extracting data, and assessment of the reporting quality of the identified articles. In addition, we used multiple keywords when retrieving literature in order to broaden the scope of the retrieved literature.

## 5. Conclusions

This review mainly provides insights into the related present situation of assessment tools for health literacy among the general population from several perspectives, such as theoretical basis, methods, domains, items, and reliability. Particularly, this review critically discusses all aspects of measuring tools and examines the reporting qualities of the identified articles to provide direction toward the further evolution of comparable and reliable health literacy assessment tools for the general population.

## Figures and Tables

**Table 1:** Main instrument characteristics of tools assessing health literacy among the general population in this literature review.

Author, Year	Scale Names	Nation	Theoretical Basis	Methods	Sample	Domains, Items (*#*)	Feasibility; Reliability; Validity	Domains
**Measurement modes based on health-related fields**								
Pleasant, A., 2008 [32]	The public health literacy knowledge scale	The United States	Thirteen essential Facts for Life messages	Expert consultation; participant feedback; Flesch–Kinkaid readability assessment	829 public (Mexico = 200, China = 220, Ghana = 204, India = 205)	13, 17	75% response rate; Cronbach’s alpha = 0.797; the public health knowledge scale and the science literacy scale = 0.391	Timing of births; safe motherhood; child development and early learning; breastfeeding, nutrition and growth; immunization; diarrhea; coughs, colds and more serious illnesses; hygiene; malaria; HIV/AIDS; injury prevention; disasters and emergencies
O’Connor, M., 2015 [33]	The mental health literacy scale	Australia	Mental health literacy consists of seven attributes; Diagnostic and Statistical Manual of Mental Disorders IV TR criteria	Developed using an iterative process; a consensus by the clinical panel; feedback	372 participants	7, 35	/; Cronbach’s alpha = 0.797; /	Ability to recognize disorders; knowledge of where to seek information; knowledge of risk factors and causes; knowledge of self-treatment; knowledge of professional help available; attitudes that promote recognition or appropriate help-seeking behavior
**Measurement modes based on health-related abilities**								
Schrauben, S.J., 2017 [34]	Zambia’s health literacy scale	Zambia	The Institute of Medicine’s (IOM) definition of health literacy	Cross-sectional questionnaire; factor analysis methods	13,646 participants between the ages of 15 and 49	4, 15	/; Cronbach’s alpha = 0.68; good content validity	Capacity to interpret; capacity to obtain; capacity to understand; make appropriate health decisions
Jordan, J.E., 2013 [35]	The health literacy management scale	Australia	/	Develop conceptual framework of health literacy (in-depth interviews, concept mapping workshops); cognitive interviews; scale score and test–retest reliability calculation	542 participants	8, 29	61% response rate; Cronbach’s alpha > 0.82; /	Patient attitudes towards their health; understanding health information; social support; socioeconomic considerations; accessing general medical practitioner (GP) healthcare services; communication with health professionals; being proactive; using health information
McCormack, L, 2010 [36]	Health Literacy Skills Instrument	The United States	/	Real-world health-related stimuli (print (prose, document, or quantitative), Internet-based information seeking), cognitive interviews	1559 Knowledge Network panelists aged 18 or over	5, 25	Completion rate = 71%, took 45 min; Cronbach’s alpha = 0.86; item-total correlations of 0.40 or higher item response theory (IRT) discrimination parameters of 1.00 or higher	Identifying and understanding health-related text; interpreting information and/or data in the form of tables, charts, pictures, symbols, maps, and videos; completing computations; making inferences based on the information presented or applying information to a specific scenario; utilizing the Internet/computer to obtain health information
Haghdoost, A.A., 2015 [37]	The Iranian Health Literacy Questionnaire	Iran	Priorities in accordance with Iranian health policies and culture sensitivity	Comprehensive review of the literature; expert consultation (health educator, an epidemiologist, and two specialists in oral health and community medicine)	1080 participants aged 18 to 60 years	10, 36	91% response rate; Cronbach’s alpha = 0.71–0.96; Kaiser–Meyer–Olkin (KMO) = 0.95, Bartlett’s test = 3.017	Reading/comprehension skills; individual empowerment (first aid skills); communication/decision making skills; assessment skills of health information in virtual media; accurate assessment/judgment skills; social empowerment; individual empowerment (household medical equipment use); health information access; health information use; health knowledge
Chinn, D., 2013 [38]	All Aspects of Health Literacy Scale	The UK	Nutbeam’s health literacy theory (functional, communicative, and critical health literacy)	Undertook a review of published research on health literacy definitions and concepts, and on its measurement; drew up a list of potential items; the course of a local consultation exercise	146 participants	3, 14	Took approximately 7 min on average; Cronbach’s alpha = 0.75; functional health literacy and communicative health literacy = 0.393, functional health literacy and critical health literacy = 0.59, communicative health literacy and critical health literacy = 0.186	Functional health literacy; communicative health literacy; critical health literacy
Suka, M., 2013 [39]	The 14-item health literacy scale	Japan	Ishikawa and colleagues’ health literacy scale specific to diabetes patients	Questionnaire	1507 eligible respondents aged 30–69 years	3, 14	96.4%–99.5% response rate; Cronbach’s alpha = 0.83; Acceptable fit of the three-factor model (comparative fit index = 0.912, normed fit index = 0.905, root mean square error of approximation = 0.082)	Functional health literacy; communicative health literacy; critical health literacy
**The combination of both measurement modes**								
Tsai, T.I., 2011 [40]	The Mandarin Health Literacy Scale	China (Taiwan)	The Institute of Medicine’s definition of health literacy (four kinds of abilities); an individual often encounters six main types of health information and health services in a health care system; three domains of literacy skills	Semi-structured in-depth interviews of health care consumers; consultation with health care, education, and psychometrics experts; generation of an item pool; selection of items for inclusion in the Mandarin Health Literacy Scale; evaluation of readability	323 Taiwanese adults	5, 50	72.1% response rate; Cronbach’s alpha = 0.97; an item-total correlation equal to or greater than 0.40	Years of schooling; reading habit; health status; health knowledge; reading assistance
Sørensen, K., 2013 [41]	The European Health Literacy Survey Questionnaire	Netherlands	A conceptual model and definition	Item development, pre-testing, field-testing, external consultation, plain language check, and translation from English to Bulgarian, Dutch, German, Greek, Polish, and Spanish	19 focus group sample, 99 pre-test sample	12, 47	Less than 95% response rate took 20–30 min; Cronbach’s alpha = 0.51–0.91; /	The three domains: healthcare; disease prevention; health promotion four-component structure: accessing; understanding; appraising and applying health related information
Intarakamhang, U., 2016 [42]	ABCDE (alcohol, baccy, coping, diet, and exercise)-health literacy scale	Thailand	The concepts of ABCDE behavior; the principles of promoting diet, managed exercise, reducing alcohol consumption, and ceasing smoking	Qualitative research methods focused on theoretical publications; expert consultation; focus groups; the causal models for measuring health literacy	4401 participants aged >15 years	8, 64	97.8% response rate; Cronbach’s alpha = 0.611–0.912; /	Needed health knowledge and understanding; accessing information and services; communicating with professionals; managing their health condition; getting media and information literacy; making appropriate health decisions to good practice; participating in social health literacy; maintaining healthy behavior

Note: # number of Dimensions and Items. / not always available.

**Table 2:** The quality of the survey studies in the development and verification of health literacy instruments.

Reporting Item	Described		Not described	
	*N*	%	*N*	%
**Background**				
Background literature review	10	90.9	1	9.1
Explicit research question	9	81.8	2	18.2
Clear study objectives *	11	100	0	0.0
**Methods**				
Description of methods of data analysis *	11	100	0	0.0
Method of questionnaire administration	1	9.1	10	90.9
Location of data collection *	11	100	0	0.0
Dates of data collection	5	45.5	6	54.5
Description of methods for replication	9	81.8	2	18.2
Methods for data entry	2	18.2	9	81.8
**Sample selection**				
Sample size calculation	0	0.0	11	100.0
Representativeness of the sample	2	18.2	9	81.8
Method of sample selection	7	63.6	4	36.4
Population and sample frame	10	90.9	1	9.1
**Research tool**				
Description of the research tool *	11	100	0	0.0
Development of research tool *	11	100	0	0.0
Instrument pretesting *	11	100	0	0.0
Instrument reliability and/or validity *	11	100	0	0.0
Scoring methods	7	63.6	4	36.4
**Results**				
Results of research presented *	11	100	0	0.0
Results address objectives *	11	100	0	0.0
Generalizability	5	45.5	6	54.5
**Response rates**				
Response rate stated	10	90.9	1	9.1
Response rate calculated	4	36.4	7	63.6
Discussion of nonresponse bias	3	27.3	8	72.7
Missing data	4	36.4	7	63.6
**Interpretation and discussion**				
Interpret and discuss findings *	11	100	0	0.0
Conclusions and recommendations *	11	100	0	0.0
Limitations	7	63.6	4	36.4
**Ethics and disclosure**				
Consent	6	54.5	5	45.5
Sponsorship	6	54.5	5	45.5
**Mean reporting frequency**		66.3		33.7

Note: * reporting item was appropriately described.

## Supplementary Materials

The following are available online at http://www.mdpi.com/1660-4601/15/8/1711/s1, Supplementary Materials S1 and Supplementary Materials S2.
