# Supplementary material for: Assessment Tools for Health Literacy among the General Population: A Systematic Review
Source: Int J Environ Res Public Health. 2018 Aug 10;15(8):1711. doi: 10.3390/ijerph15081711 (PMC6122038; doi:10.3390/ijerph15081711)
Supplement: Supplementary file 1 [file ijerph-15-01711-s001.pdf]

Supplementary Materials S1. Specific items of assessment tools for the health literacy among the public in this literature review.

Author, year	Scale Names	Domains	Items	Criterion
**measurement modes based on multiple fields**				
Pleasant, A,2008 [32]	A public health literacy knowledge scale	timing of births; safe motherhood; child development and early learning; breastfeeding, nutrition and growth; immunization; diarrhea; coughs, colds and more serious illnesses; hygiene; malaria; HIV/AIDS; injury prevention; disasters; emergen-cies	For a healthy pregnancy and birth, all pregnant women should visit a health worker before the baby is born; Births that are not assisted by a skilled birth attendant are as safe as births that are assisted by a skilled birth attendant; It is normal if children below the age of 1 year weigh the same over a 2-month period; Children who are vaccinated are protected from dangerous diseases; Overall, vaccination has more risks than benefits; Children learn a lot by playing; Most injuries and accidents cannot be prevented; If a child is breathing rapidly or has difficulty breathing,the child should be taken immediately to a health-care provider; Many diseases can be prevented by washing hands before touching food; Using condoms when having sex can prevent the spread of AIDS; Using mosquito nets helps prevent malaria; Exercise helps prevent heart disease; Coughs and colds only get better with medicine; It is the father's gene that decides whether the baby is a boy or a girl; Antibiotics kill viruses as well as bacteria; Cigarette smoking causes lung cancer; All bacteria are harmful to humans	response:True or False
O'Connor, M., 2015 [33]	The mental health literacy scale	ability to recognise disorders; knowledge of where to seek information; knowledge of risk factors and causes; knowledge of self-treatment; knowledge of professional help available; attitudes that promote recognition or appropriate help-seeking behaviour	/	mean score for the scale was 127.38 (SD¼12.63, Minimum ¼92.00, Maximum¼155.00, 95% CI¼126.09–128.67).
**measurement modes based on abilities**				

Sarah J. Schrauben, 2017 [34]	Zambia's health literacy scale	capacity to interpret; Capacity to obtain; capacity to understand; make appropriate health decisions	primary school education; Able to read whole sentence or parts; Read magazine at least once per week; Listen to radio at least once per week; Watch TV at least once per week; Heard family planning information from magazine, radio, TV in last month; Knows a place to get condoms; Knows a place to get AIDS test; Comprehensive knowledge of HIV/AIDS; Reduce chance of AIDS by always using condoms during sex; Reduce chance of AIDS by having only one sex partner with no other partner; A healthy person can have AIDS; Cannot get AIDS from mosquito bite; Cannot get AIDS from witchcraft or supernatural means; Comprehensive knowledge of HIV/AIDSb	/
Jordan, J. E., 2013 [35]	The health literacy management scale	patient attitudes towards their health; understanding health information; social support ; socioeconomic considerations; accessing GP healthcare services; communication with health professionals; being proactive; using health information	exemplar items：Make time for things that are good for your health; Pay attention to your health needs; Read written information e.g. leaflets given to you by a doctor; Ask someone to go with you to a medical appointment; Pay to see a doctor; Afford transport to medical appointments; Know where a doctor can be contacted; Know how to get a doctor's appointment; Ask a doctor questions to help you understand health information Get the information you need when seeing a doctor; Change to a different doctor to get better care; Get a second opinion about your health from a health professional; Use information from a doctor to make decisions about your health Follow instructions that a doctor gives you	response:A five-point Likert-style response format was developed in response to the content of the items: 1 = unable to do, 2 = very difficult, 3 = with some difficulty, 4 = little difficulty, 5 = without difficulty.
McCormack, L, 2010 [36]	Health Literacy Skills Instrument	identifying and understanding health-related text; interpreting information and=or data in the form of tables, charts,pictures, symbols, maps,and videos; completing computations; making inferences based on the information presented or applying information to a specific scenario; utilizing the Internet=computer to	which of the following is probably not a second-degree burn; Which of the following is a symptom of lactose intolerance; Which of the following is not a sign of a stroke; Which set of low density lipoprotein (LDL) and high density lipoprotein (HDL) levels is best; Which of the following problems could be caused by this medicine; Which of the following entrances is closest to the elevator; In the example listed in the first row of the table, when should the medicine be taken; How much will the insurance company pay for the physical therapy received on 7=22=09; How much does the patient have to pay for the laboratory services received on 7=15=09; More men die from prostate cancer than from other causes. Would you say this is true, false, or are you not sure;Who is more likely to die of	response:Choice question

		obtain health information	prostate cancer; How many grams of fiber are in two servings; If a person is on a 2,500 calorie diet, what percent of the daily value of saturated fat would he get from one serving; If a person is at high risk for heart disease, which of the following levels of low density lipoprotein (LDL) cholesterol is best; If Joe was visiting someone in Room 130 and wanted to go to the cafeteria, which of these places would he pass if he took the shortest route; A person is making a salad and wants to add one serving of chopped, uncooked carrots. How much should she use; A person is cooking dinner for himself and wants to include one serving from the meat and beans group. What should he choose; What do the muscles in the throat typically do when a person is sleeping; What part of the body do lunge exercises work; If a person was worried about his cough, what number should he press; If a person wanted to check on the date and time of an appointment she already made, what number should she press; John weighs 200 pounds and he walked at a medium pace on a firm surface for 30 minutes. How many calories did he burn; Kate weighs 150 pounds. Which activity would burn the most calories; What does this (heart attack risk) tool do; John is 39 years old and smokes. His blood pressure is 130=90 and he's on blood pressure medicine. His HDL cholesterol is 50 and his total cholesterol is 230. What is his estimated 10-year risk of heart attack	
Haghdoost, A., 2015 [37]	The Iranian Health Literacy Questionnaire	reading/comprehension skills; individual empowerment (first aid skills); communication/decision making skills; assessment skills of health information in virtual media; accurate assessment/judgment skills; social empowerment; individual empowerment (household medical Equipment use) ; health information access; health information use;	the ability to read written materials (books, etc.) on health and disease; The ability to read the written instructions of health professionals; The ability to fill in the forms requested by health centers; The ability to read guide boards in health centers; The ability to read written worksheets before performing lab tests/ultrasound/radiology; The ability to understand the concepts of guide boards; The ability to understand the concepts of guide worksheets before performing lab tests/ ultrasound/radiology; The ability to understand the conversations of health experts presented on mass media; The ability to understand the contents presented in health educational materials (newspapers, pamphlets and so on.); The ability to understand drug usage prescribed by the company or pharmacist on the package; The ability to understand health care worker's recommendations; The ability to complete forms asked by	/

health knowledge	health care centers, independently; The ability to understand Internet and electronic resources related to health and disease; Evaluating the health information presented on the Internet; Evaluating the health information presented on the Radio or TV; Evaluating the recommendations presented by physicians and/or health care workers; Evaluating health information presented on handbooks, educational pamphlets and so on; Evaluating health information presented in newspapers, journals and magazines; Evaluating recommendations presented by friends and relatives on health and disease; The ability to transfer their own health information to the others; Using prescribed antibiotics completely in spite of resolved symptoms; Refer to physician for check up in the case of having close relatives with cancer; Refer to physician for annual checkup even if there is no symptoms for having cancer; Take care of own health in any situation 0.796; The ability to communicate with health workers and ask them to re-explain their; recommendations in the case of not understanding the meaning; The ability to measure blood pressure by sphygmomanometer; The ability to measure blood sugar and applying a glucose-meter at home; The ability to measure body temperature by applying a thermometer; The ability to measure heart beat rate; The ability to perform IM injections; The ability to perform IV injections; The ability to perform first aid in the case of emergency situations; The ability to help victims in the case of an accident prior to arrival of emergency aids; Participation in public festivals such as public walking or environmental cleanup activities; Participation in the meetings of local health centers in the case of invitation; Paying attention to health priorities of political candidates while voting;

Chinn, D., 2013 [38]	All Aspects of Health Literacy Scale	functional health literacy; communicative health literacy; critical health literacy	how often do you need someone to help you when you are given information to read by your doctor, nurse or pharmacist; When you need help, can you easily get hold of someone to assist you; Aggregate of Q1 and Q2; Do you need help to fill in official documents; When you talk to a doctor or nurse, do you give them all the information they need to help you; When you talk to a doctor or nurse, do you ask the questions you need to ask; When you talk to a doctor or nurse, do you make sure they explain anything that you do not understand; Are you someone who likes to find out lots of different information about your health; How often do you think carefully about whether health information makes sense in your particular situation; How often do you try to work out whether information about your health can be trusted; Are you the sort of person who might question your doctor or nurse's advice based on your own research; Do you think that there plenty of ways to have a say in what the government does about health; Within the last 12 months have you taken action to do something about a health issue; What do you think matters most for everyone's health	response:a 3-point scale featuring the prompts rarely", "sometimes" and "often"; Single choice question.
Suka, M., 2013 [39]	The 14-item health literacy scale	functional health literacy; communicative health literacy; critical health literacy	find characters that I cannot read; Feel that the print is too small for me to read; Feel that the content is too difficult for me to understand; Feel that it takes a long time to read them; Need someone to help me read them; Collect information from various sources; Extract the information I want; Understand the obtained information; Communicate my opinion about my illness; Apply the obtained information to my daily life; Consider whether the information is applicable to me; Consider whether the information is credible; Check whether the information is valid and reliable; Collect information to make my healthcare decisions;	response: a Likert-type scale ranging from "strongly disagree"," disagree", "not sure", "agree"or "strongly agree".<br>Higher scores indicate a better HL
**the combination of both measurement modes**				

TZU-I TSAI, 2011 [40]	The Mandarin Health Literacy Scale	years of schooling; reading habit; health status; health knowledge; reading assistance	/	we determined the following ranges of health literacy levels: inadequate (0–30), marginal (31–42), and adequate (43–50).
Sørensen, K., 2013 [41]	The European Health Literacy Survey Questionnaire	the three domains: healthcare; disease prevention; health promotion<br>four-component structure: accessing; understanding; appraising and applying health related information	/	response: a Likert-type scale ranging from “very easy”,“easy”, “difficult” or “very difficult”.
Intarakamhang, U., 2016 [42]	ABCDE (alcohol, baccy, coping, diet, and exercise)-health literacy scale	needed health knowledge and understanding; accessing information and services; communicating with professionals; managing their health condition; getting media and information literacy; making appropriate health decisions to good practice; participating in social health literacy; maintaining healthy behavior	/	response: Likert(5-point scale)<br>**the score based on 3 levels of ABCDE-HL:**<br>middle level<br>high level<br>low level

Notes: / not described

Supplementary Materials S2. Specific item of the quality of the survey studies on the development and verification of health literacy instruments.

Reporting Item	Pleasant, A,2008 [32]	O'Connor, M., 2015 [33]	Sarah J. Schrauben, 2017 [34]	Jordan, J. E., 2013 [35]	McCormack, L, 2010 [36]	Haghdoost, A., 2015 [37]	Chinn, D., 2013 [38]	Suka, M., 2013 [39]	TZU-I TSAI, 2011 [40]	Sørensen, K., 2013 [41]	Intarakamhang, U., 2016 [42]
**Background**											
Background literature review	*	*	*	/	*	*	*	*	*	*	*
Explicit research question	*	*	/	*	*	*	/	*	*	*	*

Clear study objectives	*	*	*	*	*	*	*	*	*	*	*
**Methods**											
Description of methods of data analysis	*	*	*	*	*	*	*	*	*	*	*
Method of questionnaire administration	/	/	*	/	/	/	/	/	/	/	/
Location of data collection	*	*	*	*	*	*	*	*	*	*	*
Dates of data collection	*	/	*	/	*	*	/	*	/	/	/
Description of methods for replication	*	*	/	*	*	*	/	*	*	*	*
Methods for data entry	/	/	/	*	/	*	/	/	/	/	/
**Sample selection**											
Sample size calculation	/	/	/	/	/	/	/	/	/	/	/
Representativeness of the sample	*	/	/	/	/	/	/	/	/	/	*
Method of sample selection	/	/	*	*	*	*	*	*	*	/	/
Population and sample frame	*	*	*	*	*	*	*	*	*	*	/
**Research tool**											
Description of the research tool	*	*	*	*	*	*	*	*	*	*	*

development of research tool	*	*	*	*	*	*	*	*	*	*	*
Instrument pretesting	*	*	*	*	*	*	*	*	*	*	*
Instrument reliability and/or validity	*	*	*	*	*	*	*	*	*	*	*
Scoring methods	/	*	/	*	/	/	*	*	*	*	*
**Results**											
Results of research presented	*	*	*	*	*	*	*	*	*	*	*
Results address objectives	*	*	*	*	*	*	*	*	*	*	*
Generalisability	*	*	*	/	/	/	*	*	/	/	/
**Response rates**											
Response rate stated	*	*	/	*	*	*	*	*	*	*	*
Response rate calculated	/	/	/	*	*	*	/	*	/	/	/
Discussion of nonresponse bias	/	/	/	/	*	*	/	/	*	/	/
Missing data	/	/	/	*	*	*	/	*	/	/	/
**Interpretation and discussion**											
Interpret and discuss findings	*	*	*	*	*	*	*	*	*	*	*

Conclusions and recommendations	*	*	*	*	*	*	*	*	*	*	*
Limitations	/	*	*	*	/	*	/	*	*	*	/
**Ethics and disclosure**											
Consent	*	/	*	*	/	*	/	*	/	/	*
Sponsorship	*	/	*	/	/	*	/	*	/	/	*

Notes: (1) * described; (2) / not described
